# Integration of Transcriptomes, Small RNAs, and Degradome Sequencing to Identify Putative miRNAs and Their Targets Related to Eu-Rubber Biosynthesis in *Eucommia ulmoides*

**DOI:** 10.3390/genes10080623

**Published:** 2019-08-19

**Authors:** Jing Ye, Wenjing Han, Ruisheng Fan, Minhao Liu, Long Li, Xiaoming Jia

**Affiliations:** College of Forestry, Northwest A&F University, Shaanxi 712100, China

**Keywords:** microRNA, transcriptome, degradome, high-throughput sequencing, Eu-rubber, *Eucommia ulmoides*

## Abstract

*Eucommia ulmoides* has attracted much attention as a valuable natural rubber (Eu-rubber) production tree. As a strategic material, Eu-rubber plays a vital role in general and defence industries. However, the study of Eu-rubber biosynthesis at a molecular level is scarce, and the regulatory network between microRNAs (miRNAs) and messenger RNAs (mRNAs) in Eu-rubber biosynthesis has not been assessed. In this study, we comprehensively analyzed the transcriptomes, small RNAs (sRNAs) and degradome to reveal the regulatory network of Eu-rubber biosynthesis in *E. ulmoides*. A total of 82,065 unigenes and 221 miRNAs were identified using high-throughput sequencing; 20,815 targets were predicted using psRNATarget software. Of these targets, 779 miRNA-target pairs were identified via degradome sequencing. Thirty-one miRNAs were differentially expressed; 22 targets of 34 miRNAs were annotated in the terpenoid backbone biosynthesis pathway (ko00900) based on the Kyoto Encyclopedia of Genes and Genomes (KEGG). These miRNAs were putatively related to Eu-rubber biosynthesis. A regulatory network was constructed according to the expression profiles of miRNAs and their targets. These results provide a comprehensive analysis of transcriptomics, sRNAs and degradome to reveal the Eu-rubber accumulation, and provide new insights into genetic engineering techniques which may improve the content of Eu-rubber in *E. ulmoides*.

## 1. Introduction

MicroRNAs (miRNAs) are endogenous, non-coding, regulatory small RNAs (sRNAs) that are widely distributed in the genome of animals and plants at 18–25 nt in length [1]. miRNAs are a class of regulatory factors of eukaryotic gene expression, mainly by guiding cleavage or translational reduction of targets to regulate gene expression at transcriptional or post-transcriptional levels [2,3]. In previous reports, miRNAs have played critical roles in plant growth and development, flowering timing, metabolic processes, and abiotic and biotic stress responses [4,5,6]. Furthermore, the terpenoid and flavonoid biosyntheses were also regulated by miRNAs [7,8,9,10]. In *Hevea brasiliensis* (rubber tree), miR159b was inferred to be involved in latex biosynthesis [11], and the expression level of hbr-miR172 significantly affected the content of latex [12]. These findings indicated that some miRNAs play key roles in rubber’s biosynthesis.

*Eucommia ulmoides* is an important economic tree species of Eucommiaceae [13]. It has attracted much attention as a valuable natural rubber tree [14,15,16]. Due to the advent of the Quaternary Ice Age, only a few *E. ulmoides* survived in China. Currently, *E. ulmoides* is naturally distributed in temperate and subtropical regions of China (24°50′ N–41°50′ N, 76°00′ E–126°00′ E) in 27 provinces, and its cultivated area it was introduced within is 0.35 million hectares [13]. *E. ulmoides* has superior temperature tolerance and soil tolerance; it can withstand temperatures from 30 °C to 44 °C and normally grows in poor soil [13]. In addition, *E. ulmoides* also has a strong drought tolerance [13].

Natural rubber is an isoprenoid polymer. In 2015, the global demand for natural rubber reached 12.14 million tons, and demand is still growing steadily [17]. As the main source of natural rubber, rubber trees are vulnerable to pests and diseases and have a narrow distribution range, which causes enormous challenges for the rubber industry [18,19,20]. *Eucommia* rubber (Eu-rubber) is a trans-polyisoprene with the dual properties of rubber and plastic. The new material developed as Eu-rubber has the advantages of acid- and alkali-corrosion resistance and high insulation [21]. *E. ulmoides* has strong environmental adaptability, wide planting areas, and high-quality Eu-rubber, which makes up for the deficiency of the rubber tree. Therefore, *E. ulmoides* is an ideal substitute plant for harvesting natural rubber. However, studies on Eu-rubber biosynthesis at the molecular level are very limited, and the regulatory network between miRNAs and messenger RNAs (mRNAs) in Eu-rubber biosynthesis has not been fully described.

In this study, to reveal the molecular mechanism of Eu-rubber biosynthesis, the genome-wide regulatory network between miRNAs and mRNA was studied. *E. ulmoides* leaves with significant differences in Eu-rubber content were used to construct mRNA libraries and sRNA libraries, and their total RNAs were used in equal amounts to construct a degradome library. The Illumina system was employed for sequencing. The results of this study will allow us to further understand the regulatory network of terpenoid biosynthesis in plants and provide new insights into the use of genetic engineering techniques to improve the content of Eu-rubber in *E. ulmoides.*

## 2. Materials and Methods

### 2.1. Plant Materials and Growth Conditions

*E. ulmoides* plants used in this study were planted in the nursery of the College of Forestry, Northwest A&F University (Yangling, Shaanxi, China) with well management. The leaves of *E. ulmoides* were collected on 13 October 2016 for sequencing analysis. The samples were labeled LR (low Eu-rubber content, 1.39%, the average rate of Eu-rubber) and HR (high Eu-rubber content, 4.80%). Each sample was pooled from three individual plants, and three biological replicates were collected. The samples were frozen in liquid nitrogen immediately after collection, then stored at −80 °C before use.

### 2.2. Total RNA Preparation

Total RNA from six samples (LR01, LR02, LR03 and HR01, HR02, HR03) was extracted using TRIzol reagent (Invitrogen, CA, USA) according to the manufacturer’s instructions. The concentration and integrity of the total RNA were analyzed by Qubit^®^ RNA Assay Kit in Qubit^®^2.0 Fluorometer (Life Technologies, CA, USA) and RNA Nano 6000 Assay Kit of the Agilent Bioanalyzer 2100 system (Agilent Technologies, CA, USA), respectively. The purified RNA was used to construct transcriptome libraries, sRNA libraries, and a degradome library.

### 2.3. Transcriptome Sequencing and De Novo Assembly Analysis

mRNA was purified from total RNA using poly-T oligo-attached magnetic beads. Then, the NEBNext First Strand Synthesis Reaction Buffer (5x) was used to fragment. The first strand cDNA was synthesized using random hexamer primer and M-MuLV Reverse Transcriptase (RNase H-). The second strand cDNA synthesis was subsequently performed using DNA Polymerase I and RNase H. Remaining overhangs were converted into blunt ends via exonuclease/polymerase activities. After adenylation of 3′ ends of DNA fragments, NEBNext Adaptors with hairpin loop structures were ligated to prepare for hybridization. After purification by the AMPure XP system (Beckman Coulter, Beverly, USA) and PCR enrichment, the final products were subjected to transcriptome sequencing using an Illumina HiSeq X-ten platform. The sequencing data were submitted and deposited at the National Center for Biotechnology Information (NCBI) Sequence Read Archive—the accession number is SRP158357.

After filtering the primer, adaptor, and low-quality sequence from the total reads, the remaining clean reads were used for de novo transcriptome assembly by Trinity software [22]. Unigenes were further classified and annotated based on existing databases.

### 2.4. sRNA Sequencing and miRNAs Identification

The sRNA library construction was performed using the NEB Next Ultra small RNA Sample Library Prep Kit for Illumina (New England Biolab, MA, USA) according to the manufacture’s protocols. Briefly, T4 RNA ligase was used on the adapters at the 3′ and 5′ ends of the sRNA, respectively. Subsequently, the sRNA with adapters was reverse transcribed into cDNA. Then, PCR enrichment and 18–30 nt of small RNAs were recovered with 12% PAGE gel. Finally, the purified products were subjected to sRNA sequencing using an Illumina HiSeq2500 platform. The sequencing data were submitted and deposited at the NCBI Sequence Read Archive, with the accession number SRP216820.

High-quality reads were generated by removing the reads with “N” content ≥ 10%, the reads without the 3′-adaptor, adaptor sequences, low-quality reads, and sequences shorter than 18 nt or longer than 30 nt. In order to classify the clean reads, Bowtie [23] software was employed to search the clean reads against a series of databases, including Silva, GtRNAdb, and Repbase database. After filtering out the ribosomal RNA (rRNA), transport RNA (tRNA), nuclear small RNA (snRNA), nucleolar small RNA (snoRNA) and repeat sequences, the unannotated reads were mapped to the reference genome of *E. ulmoides* using the Bowtie software. The perfect match sequences were retained for further analysis. These mapped sequences were aligned with a mature sequence of known miRNAs in the miRBase database (version 22.1) (http://www.mirbase.org/) with a maximum of three mismatches allowed to identify known miRNAs of *E. ulmoides*. Novel miRNAs were predicted from unannotated sRNA sequences using miRDeep2 software [24]. The basic criteria were used to screen for candidate new miRNAs [25]. Because of the small difference in length of miRNAs, we quantified the transcript levels using transcripts per million (TPM) according to the normalization formula: TPM = Mapped read count/ Total reads × 1,000,000 [10].

### 2.5. Degradome Sequencing and Target Identification and Prediction

The total RNA of these six samples was mixed in equal amounts to construct a degradome library. The Oligotex mRNA mini kit (Qiagen, Hilden, Germany) was employed to isolate the Poly(A) RNA from the total RNA. The method of constructing the degradome library is as follows: Polyadenylated transcripts possessing 5′-monophosphates were ligated to an RNA oligonucleotide adaptor containing a *MmeI* recognition site using T4 DNA ligase, first-strand cDNA was synthesized, and finally, cDNA was PCR-enriched, gel-purified, and sequenced using an Illumina HiSeq2500 platform. The sequencing data were submitted and deposited at the NCBI Sequence Read Archive, with the accession number SRP216712.

After removing the adaptor sequences and the low-quality sequences, only 20 or 21 nt high-quality sequences were used for subsequent analysis. The degradome reads were mapped to the reference genome. Perfectly matched sequences were used as candidate sequences to predict the potentially cleaved targets using CleaveLand pipeline [26]. A maximum of five mismatches was allowed, and no mismatches were allowed at the cleavage sites between the 10^th^ and 11^th^ nucleotides. The identified targets were classified into different categories based on the previous criteria [27].

In addition, psRNATarget (2017 update) [28] software was used to predict targets for all miRNAs. The schema V2 (2017 release) was chosen, and the parameters were the default values.

### 2.6. Functional Annotation of Targets

To determine the function of targets, gene ontology (GO) and Kyoto Encyclopedia of Genes and Genomes (KEGG) enrichment were carried out. GO enrichment analysis was performed using the web-based agriGO program (GO Enrichment Analysis Software Toolkit and Agricultural Community) [29]. The targets were searched against *Arabidopsis* protein sequences (ftp://ftp.arabidopsis.org/Proteins/TAIR10_protein_lists/TAIR10_pep_20101214) using BLASTX to obtain TAIR10 locus IDs, which were submitted to agriGO as a query. The enriched GO terms were analyzed using default parameters. The terms with corrected *p*-values < 0.05 were defined as significantly enriched terms. Then, the significantly enriched GO terms were visualized and plotted using ReviGO tool (http://revigo.irb.hr/) [30] with the default parameters. Furthermore, the targets were mapped to their respective pathways using KEGG Automatic Annotatioc [31]. Pathway enrichment analysis was done based on the hypergeometric model [32] with a significance threshold of *p*-value 0.05.

### 2.7. RLM-5′RACE to Verify the Identified and Predicted Targets

To map the cleavage site of target transcripts, RNA ligase-mediated rapid amplification of 5′ cDNA ends (RLM-5′RACE) was performed using a FirstChoice^®^ RLM-RACE Kit (Invitrogen, CA, USA) according to the manufacturer’s protocols [33]. In brief, a 5′RACE adapter was ligated to the total RNA. Subsequently, the ligated RNA was reverse transcribed. A nested PCR was performed using the gene-specific primers and universal primers (Table S1). The RACE products were purified by 2% gel, cloned, and their sequence was analyzed.

### 2.8. Validation of miRNAs’ and Their Targets’ Expression by RT-qPCR

A total of 11 differentially expressed miRNA-target pairs were randomly selected to validate the high-throughput sequencing results by real-time quantitative PCR (RT-qPCR). The RNA samples used for RT-qPCR were identical to the sequencing samples. For miRNAs, the Mir-X miRNA First-Strand Synthesis Kit (TaKaRa, Dalian, China) and Mir-X miRNA qRT-PCR SYBR Kit (TaKaRa) were employed to reverse transcribe reactions and RT-qPCR reactions. The specific forward primers for RT-qPCR were designed according to each mature miRNA sequence, a universal primer as the reverse primer, and *U6* as the internal reference gene. The PCR reaction mixture (20 μL) contained 2 μL of cDNA (ten-fold dilution), 10 μL of SYBR Advantage Premix (2×), 0.4 μL of each primer (10 nmol mL^−1^), and 7.2 μL of ddH_2_O. The amplification conditions were carried out as follows: 95 °C for 20 s, 40 cycles at 95 °C for 5 s, 58 °C for 30 s, and 72 °C for 30 s. For target genes, primer design method, reagents, reaction system, and reaction procedure, the protocols were the same as in the previous study [34]. *UBC* was the internal reference gene [34]. Each reaction was processed in triplicate. The 2^−△△Ct^ method was used to calculate the relative expression levels for miRNAs and their targets. The primers for RT-qPCR were embodied in Table S2.

## 3. Results

### 3.1. Overview of mRNAs and sRNAs Sequencing Data

Our aim was to reveal potential mechanisms of Eu-rubber biosynthesis in *E. ulmoides*. High-throughput sequencing was employed to analyze the mRNA and miRNA expression profiles in *E. ulmoides* with different Eu-rubber contents (three replicates per group; low Eu-rubber contents: LR01, LR02, LR03; high Eu-rubber contents: HR01, HR02, HR03).

For mRNA sequencing, there were 86,712,451 and 87,010,501 raw reads for LR and HR, respectively. After removing the sequencing adapters and primer sequences, and filtering out the low-quality reads, 76,378,632 and 77,164,214 clean reads were obtained, respectively. Between them, 55,114,110 (72.10%) and 53,913,610 (69.94%) reads were mapped to the reference genome, respectively (Table S3). A total of 82,065 unigenes were obtained by de novo assembly.

For sRNAs sequencing, a total of 138,362,960 raw reads were obtained from the six libraries generated from LR (72,342,259) and HR (66,020,701) (Table S4). After eliminating low-quality reads and impurities, and discarding 3′-adapter deletions, insertion deletions, 5′-adapter contaminants, poly-A sequences, and sequences less than 18 nt and more than 30 nt reads, we obtained 49,746,562 and 42,690,163 clean reads in the LR and HR libraries, respectively (Table S4). This provides a wealth of data for the further study of sRNA. In each sample, the clean reads were annotated and matched to GtRNAdb, Silva, Rfam, and Repbase databases to classify the sRNAs. Among them, rRNA accounted for the highest proportion of non-coding RNA, accounting for about one-third of this group (Table S5). The unannotated RNA accounts for the majority of the clean reads (64.43%), which includes potential miRNAs (Table S5).

**Figure 1:**
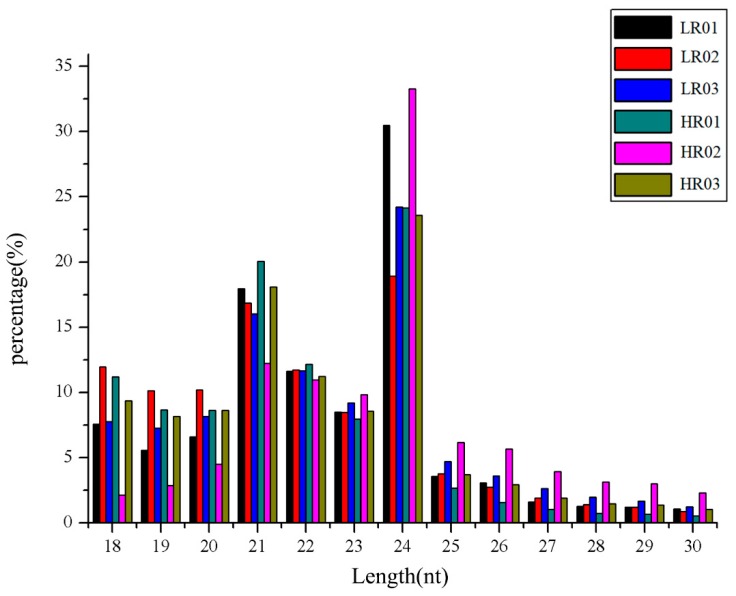
Length distribution and frequency of small RNAs (sRNAs) in the six *Eucommia ulmoides* libraries.

The size distribution of sRNAs was summarized in Figure 1. In general, most sRNAs were concentrated in the 18–24 nt range. Among these sequences, the 24 nt sRNAs accounted for the highest proportion, followed by the 21 nt sRNAs in the six libraries. After eliminating the rRNA, scRNA, snRNA, snoRNA, tRNA, and repbase, about 15% of the clean reads could be mapped to the *E. ulmoides* transcriptome (Table S6). However, most of the sequence could not be mapped because the complete genome sequence of *E. ulmoides* is not available.

### 3.2. Identification of miRNAs in *E. ulmoides*

**Figure 2:**
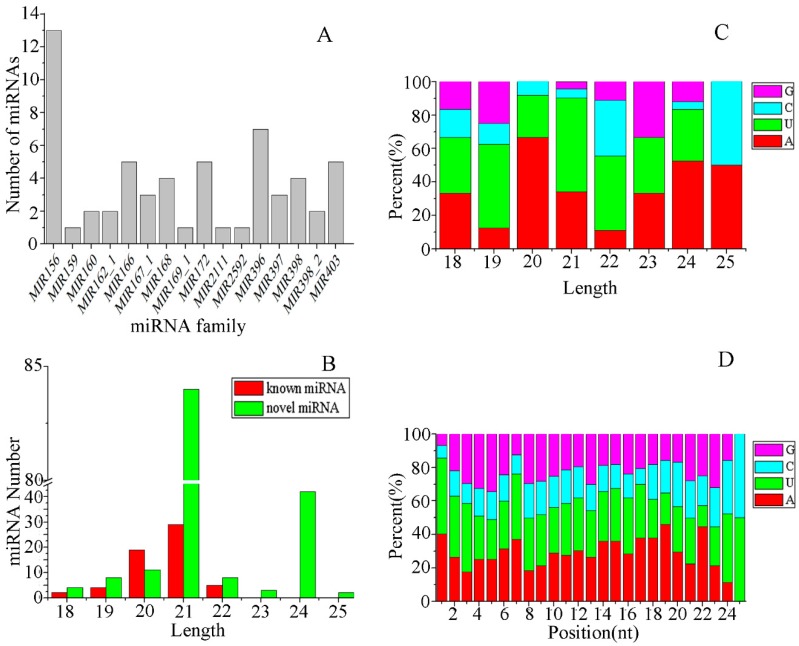
microRNA (miRNA) families, length distribution, and nucleotide bias of miRNAs in *E. ulmoides*. (**A**) Numbers of identified miRNAs in known miRNA families. (**B**) Length distribution of known miRNAs and novel miRNAs. (**C**) The first nucleotide bias of miRNAs with different lengths. (**D**) Nucleotide bias at each position in miRNAs.

It is well known that the sequence of miRNAs is highly conserved across species. To identify the miRNAs from *E. ulmoides* leaves, all unannotated sRNA sequences from six libraries were aligned against all known plant miRNAs in the miRBase database (Release 22.1) with no more than three mismatches. A total of 59 known miRNAs were identified in the six libraries, which belonged to 16 miRNA families (Figure 2A and Table S7). Among them, the number of miRNA members in each miRNA family differed substantially. There were several major miRNA families, such as MIR156, which was the largest miRNA family with 13 members, followed by MIR396 with seven members, MIR166 / 172 / 403 (five members each), MIR168 / 398 (four members each), MIR167_1 / 397 (three members each), MIR160 / 162_1 / 398_2 (two members each), and MIR159 / 169_1 / 2111 / 2592 had only one member each (Figure 2A). Most of these miRNA families were highly conserved (found in ≥ 10 species), such as MIR156 / 396 / 166 / 159 / 160 / 167_1 / 172 / 168 / 398 / 397 / 169_1 / 403 and MIR2111 (Table S8). Among them, MIR156 had the highest level of conservation and was found in 53 species, followed by MIR396 and MIR166, which were found across 46 and 45 plant species, respectively (Table S6). MIR162_1 and MIR398_2 showed low levels of conservation and were found in four and two species, respectively (Table S8). However, MIR2592 was classified as non-conserved because it was only found in *Medicago truncatula* in previous studies (Table S8). In addition, the remaining unannotated sRNA sequences were aligned with the previously published *E. ulmoides* miRNA mature sequences [10] (not included in the miRbase 22.1 database) with no mismatches. A total of nine miRNAs were matched and were also considered to be known miRNAs. The length of the known miRNAs was in the range of 18–22 nt, and it was dominant at 21 nt and 20 nt (Figure 2B).

The main difference between miRNAs and other small-molecule RNAs is that their precursors can form a signature stem-loop structure, which serves as the basis for predicting novel miRNAs. For the remaining sequences that were not identified as known miRNAs, the miRDeep2 was employed to predict novel miRNAs. In the present study, a total of 153 novel miRNAs were predicted in all sRNA libraries. The precursors of these novel miRNAs had a minimal folding free energy (MFE) between -133.6 and -15.8 kcal/mol (Table S9). The length of novel miRNAs was distributed within the range of 18–25 nt, mainly 21 nt and 24 nt (Figure 2B).

Since mature miRNAs are produced by Dicer enzyme and DCL enzyme cleavage of the precursor, the first base at the 5′ terminal has a strong preference for uracil (U). We analyzed the nucleotide bias of miRNAs. The results showed that the first nucleotide of miRNAs with different lengths has a different bias (Figure 2C). In the 18 and 25 nt miRNAs, adenine (A) and U had the same frequency. In the miRNAs with 19, 21, and 22 nt, the probability of the first nucleotide being U was much higher. However, the first nucleotide of miRNAs with a length of 20 and 24 nt tended to be A. In addition, nucleotide bias at each position was also analyzed (Figure 2D). At the first three positions, U appears most frequently; the 4^th^ and 5^th^ positions were most likely guanine (G); the 6^th^–24^th^ positions, the A, U, and G occurred randomly, the frequency of cytosine (C) was always the least in the 1^st^–23^rd^ positions; the 25^th^ position only contained U and C at the same frequency.

### 3.3. Eu-Rubber Biosynthesis-Related miRNAs

**Figure 3:**
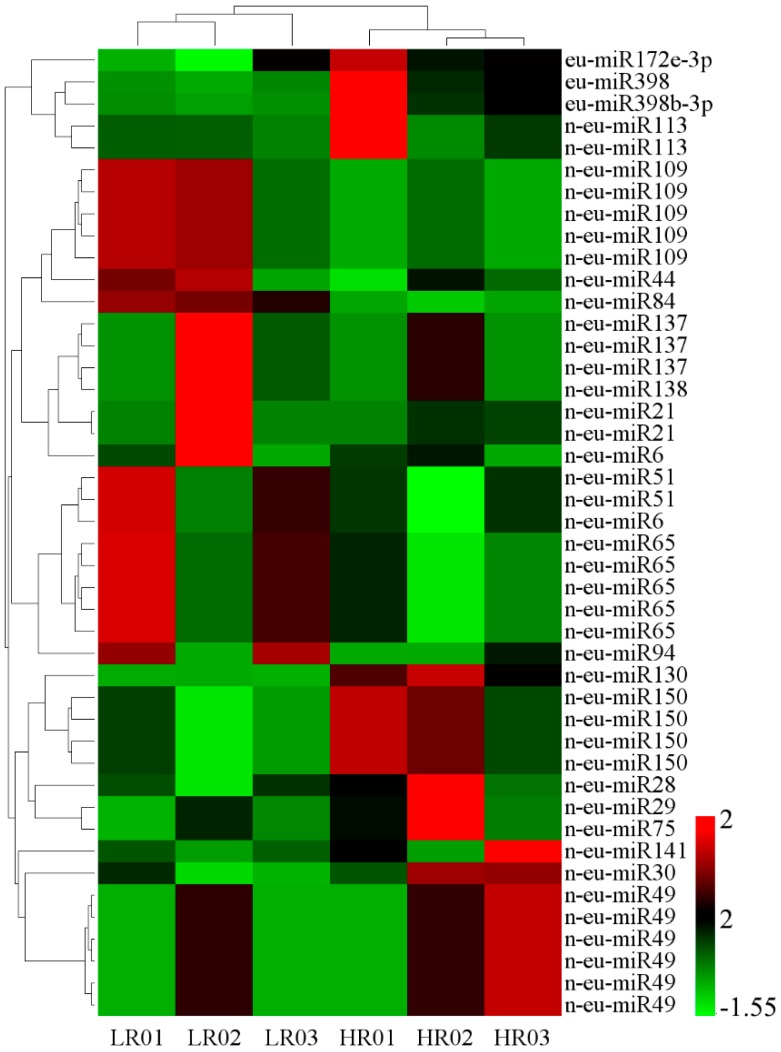
*Eucommia* rubber (Eu-rubber) biosynthesis-related miRNAs in *E. ulmoides*. Differentially expressed miRNAs (DEM) in LR (low Eu-rubber content) and HR (high Eu-rubber content) libraries by hierarchical clustering. Red indicates a high expression level and green indicates a low expression level. The original expression values of miRNAs were normalized using Z-score normalization.

To identify Eu-rubber biosynthesis-related miRNAs in *E. ulmoides*, differential expression analysis of miRNAs between LR and HR libraries was performed. The reads of all miRNAs were normalized by TPM, and differential expression analysis was performed using DESeq software with thresholds set to |log2(fold change)| ≥ 1 and false discovery rate (FDR) ≤ 0.05. A total of 31 miRNAs, including four known miRNAs belonging to two miRNA families and 27 novel miRNAs showed differential expression patterns, which were putatively related to Eu-rubber biosynthesis (Supplementary Table S10, Figure 3). According to the heatmap of differentially expressed miRNAs (DEMs) (Figure 3), 19 out of 31 miRNAs were up-regulated in the HR libraries compared with the LR libraries, including all four known miRNAs. Moreover, 12 novel miRNAs showed down-regulated patterns. The n-eu-miR130 and n-eu-miR138 were the most DEMs with ratios of 3.67-fold and 3.66-fold, respectively. These miRNAs might play important roles in Eu-rubber biosynthesis in *E. ulmoides*. The results provided an important basis for the study of miRNAs involved in the regulation of Eu-rubber biosynthesis in *E. ulmoides*.

### 3.4. A Target Prediction, Identification and Validation

**Figure 4:**
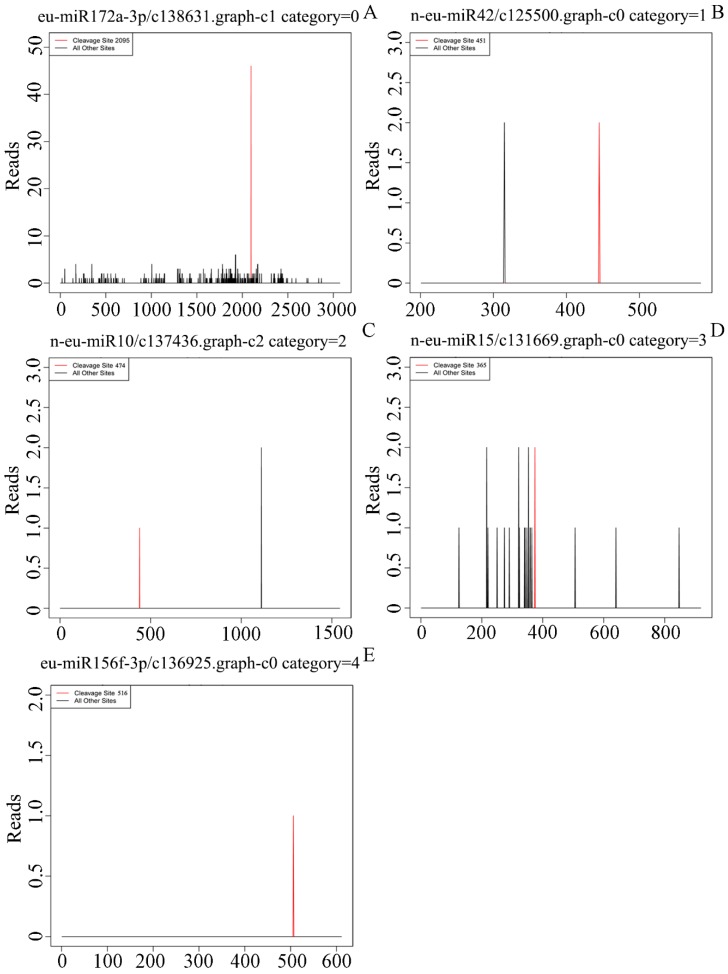
Target plots (T-plots) for miRNA targets in the five different categories validated by degradome sequencing. The x-axis indicates the site position of target cDNA, and the y-axis indicates the normal abundance of raw tags. The red line indicates the identified cleavage site. (**A**) Example of the category 0; (**B**) example of the category 1; (**C**) example of the category 2; (**D**) example of the category 3; (**E**) example of the category 4.

To investigate the diverse biological functions of miRNAs, it is particularly important to identify targets for miRNAs. In the present study, we predicted the targets for known and novel miRNAs using psRNATarget software. All miRNAs had predicted their targets; a total of 20,815 unigenes were targeted by these 221 miRNAs (Supplementary Table S11a). Furthermore, we identified the targets for miRNAs in transcriptome-wide using high-throughput degradome sequencing. After filtering the adaptor sequences and the low-quality sequences from the raw reads, a total of 24,923,386 clean reads with 8,068,041 unique reads were obtained. Among them, 4,683,286 clean reads (including 4,011,009 perfectly matched and 672,277 imperfectly matched reads) were mapped to the *E. ulmoides* reference genome. The Cleaveland 3.0 pipeline [26] was employed to identify the sliced targets for all miRNAs detected in this study. The abundance of transcripts was plotted for each transcript, and the sliced-target transcripts were grouped into five categories (0, 1, 2, 3 and 4) according to the relative abundance of tags at the target sites (Figure 4 and Supplementary Table S11b). Category ‘0′ is defined as > 1 raw read at the position, abundance at position is equal to the maximum on the transcript, and there is only one maximum on the transcript. Category ‘1′ is defined as > 1 raw read at the position, abundance at position is equal to the maximum on the transcript, and there is more than one maximum position on the transcript. Category ‘2′ is defined as > 1 raw read at the position, abundance at position is less than the maximum, but higher than the median for the transcript. Category ‘3′ is defined as > 1 raw read at the position, abundance at position is equal to or less than the median for the transcript. Category ‘4′ is defined as only one raw read at the position. Based on the degradation sequencing, a total of 779 miRNA-target pairs were identified in the prediction result, including 208 known miRNA-target pairs and 571 novel miRNA-target pairs (Supplementary Table S11b). 

In the identified miRNA-target pairs, most miRNAs only cleaved a single target transcript, and different members of the miRNA families cleaved the same target gene or different members of a gene family. For instance, six members of the MIR156 family in this study co-cleaved the same transcript (c134050.graph_c0) at the same cleavage site; three members of the MIR172 family cleaved the transcript (c138631.graph_c1) at the same cleavage site (Supplementary Table S11b). However, two or more target transcripts were identified by degradome sequencing for the same miRNAs. For instance, n-eu-miR15 had 41 target transcripts, n-eu-miR42 had 35 target transcripts, and eu-miR396h had eight target transcripts (Supplementary Table S11b). This showed that the regulation process of miRNAs in the life process was very complicated.

**Figure 5:**
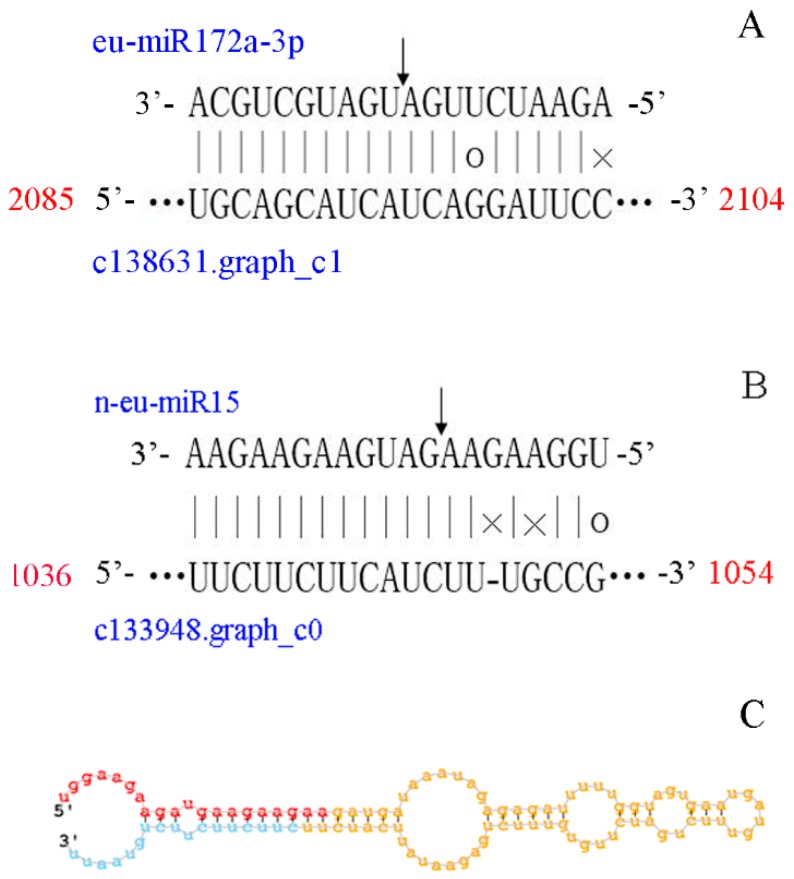
Confirmation of the cleavage sites of eu-miR172a-3p and n-eu-miR15 with their targets by RLM-5′ RACE; (**A**) eu-miR172a-3p/ c138631.graph_c1; (**B**) n-eu-miR15/ c133948.graph_c0; and (**C**) stem-loop structure of n-eu-miR15. Watson-Crick pairing (vertical dashes), G:U wobble pairing (circles), and mismatched bases (crosses) are indicated. The vertical arrows indicate the cleavage sites identified by RLM-5′ RACE. The red portion of the stem-loop structure is the mature sequence of n-eu-miR15.

To verify the targets identified by degradome sequencing and predicted by psRNATarget software, eu-miR172a-3p/ c138631.graph_c1 (identified by degradome sequencing) and n-eu-miR15/ c133948.graph_c0 (predicted by psRNATarget software) were selected for RLM-5′ RACE analysis. The cleavage site of eu-miR172a-3p/ c138631.graph_c1 pair was between the 10^th^ and 11^th^ nucleotide of eu-miR172a-3p; the same result was found with degradome sequencing (Figure 5A). Furthermore, the cleavage site of n-eu-miR15/ c133948.graph_c0 was between the 8^th^ and 9^th^ nucleotides (Figure 5B). The results of RLM-5′ RACE indicated that the targets identified by degradome and predicted by psRNATarget software were reliable.

### 3.5. Function Analysis of Targets for miRNAs in *E. ulmoides*

**Figure 6:**
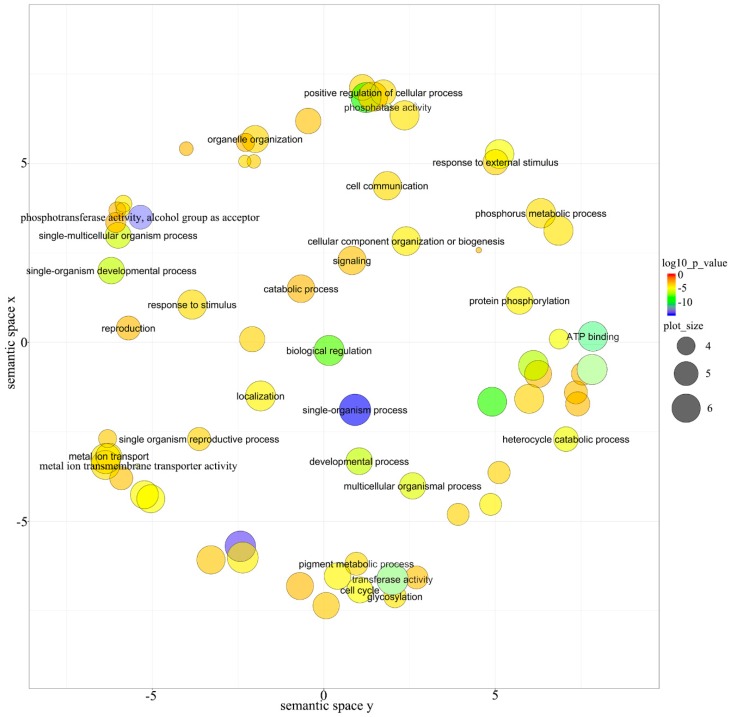
Gene ontology (GO) enrichment analysis of predicted target genes. The color indicates the *p*-values; size indicates the frequency of the GO term in the GOA database. The bubbles’ x and y coordinates were derived by applying multidimensional scaling to a matrix of the GO terms’ semantic similarities; consequently, their closeness on the plot should closely reflect their closeness in the GO graph structure, i.e., the semantic similarity.

To better understand the function of miRNAs in this study, the GO enrichment analysis was performed on targets of all miRNAs based on the GO database (http://www.geneontology.org/). A total of 129 GO terms were significantly enriched (*p*-value < 0.05, *P*. adjust < 0.05), including 119 biological process terms and 10 molecular components terms. GO terms were also grouped and visualized [30] to reveal the representative biological processes that were potentially involved in miRNAs functions (Figure 6). The targets were involved in many meaningful biological processes, including single-organism process, single-organism developmental process, positive regulation of cellular process, single-multicellular organism process, developmental process, multicellular organismal process, metal ion transport, heterocycle catabolic process, transferase activity, biological regulation, cell cycle, phosphotransferase activity, alcohol group as acceptor, pigment metabolic process, signaling, response to stimulus, response to external stimulus, ATP binding, and so on (Figure 6). The results indicated that miRNAs played important roles in the growth and development, reproduction, response to biological and abiotic stress, metabolism, metal ion transport and other processes of *E. ulmoides*.

The pathway annotation was also analyzed for miRNA targets based on the Kyoto Encyclopedia of Genes and Genomes (KEGG) database. A total of 1866 miRNA targets were assigned to 107 pathways (Supplementary Table S12). The ten pathways with the most abundant miRNA targets were Ribosome (ko03010), biosynthesis of amino acids (ko01230), Protein processing in endoplasmic reticulum (ko04141), Spliceosome (ko03040), Plant hormone signal transduction (ko04075), Plant-pathogen interaction (ko04626), Carbon metabolism (ko01200), RNA transport (ko03013), Endocytosis (ko04144), and RNA degradation (ko03018). In addition, 22 miRNA targets were annotated in the terpenoid backbone biosynthesis pathway (ko00900), which was the main pathway for Eu-rubber biosynthesis (Table 1). This indicates that miRNAs play important roles in Eu-rubber biosynthesis.

### 3.6. Correlation Analysis of miRNAs and Their Targets’ Expression Profiles

**Figure 7:**
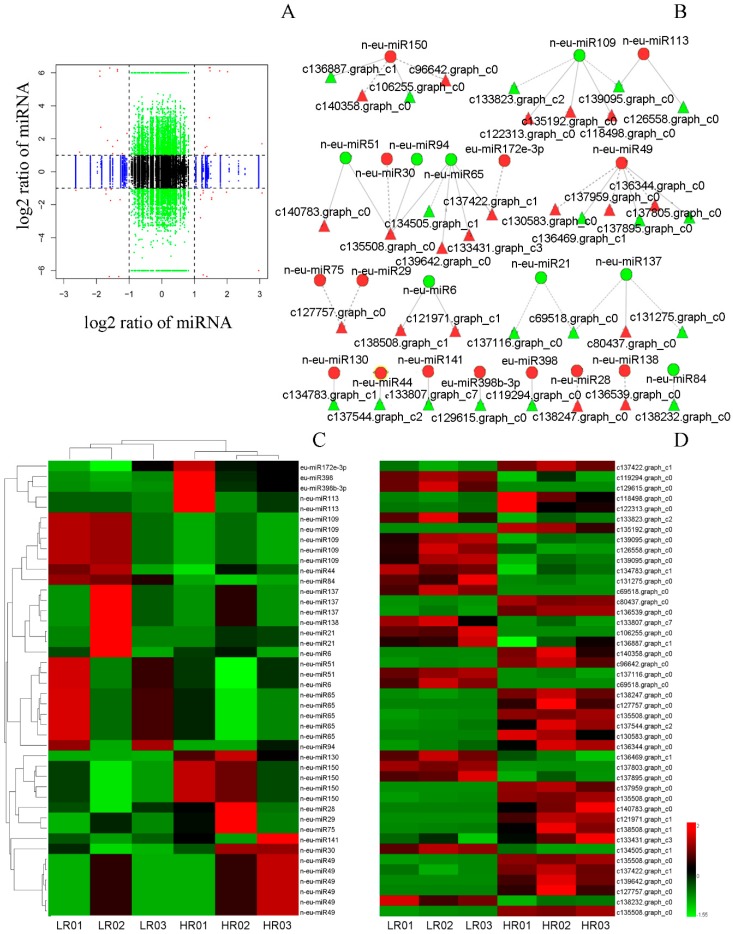
A combined view of the genome-wide expression profiles and the regulatory relationship between miRNAs and their targets in *E. ulmoides*. (**A**) Overview of genome-wide expression profiles between miRNA and their targets. The x-axis represents the miRNAs expression levels (log_2_(fold-change)), and the y-axis represents the target genes expression levels (log_2_(fold-change)); (**B**) the interaction network between differentially expressed miRNAs (DEMs) and their differentially expressed targets in *E. ulmoides*. Red circles indicate up-regulated miRNAs, green circles indicate down-regulated miRNAs, and red triangles indicate up-regulated targets, green triangles indicate down-regulated targets; (**C**,**D**) the combined view of expressions levels between DEMs and their differentially expressed targets, respectively. Both the original expression values of miRNAs and targets were normalized using Z-score normalization.

To explore the regulatory network of miRNAs and their targets in the biosynthesis of Eu-rubber in *E. ulmoides*, the genome-wide expression profiles analyses of miRNAs and transcriptomes were performed in *E. ulmoides* with different content rates of Eu-rubber (Figure 7A). By comparing transcriptome analyses, 568 differentially expressed genes (DEGs) were identified, including 341 up-regulated and 227 down-regulated unigenes. Among them, 214 DEGs were predicted to be the targets of miRNAs, and 37 DEGs were targeted by 22 DEMs, forming 44 miRNA-target pairs (Figure 7B–D). Of these, 25 miRNA-target pairs showed anti-correlation expression patterns, and 19 miRNA-target pairs showed positive correlation expression patterns (Figure 7B–D).

**Figure 8:**
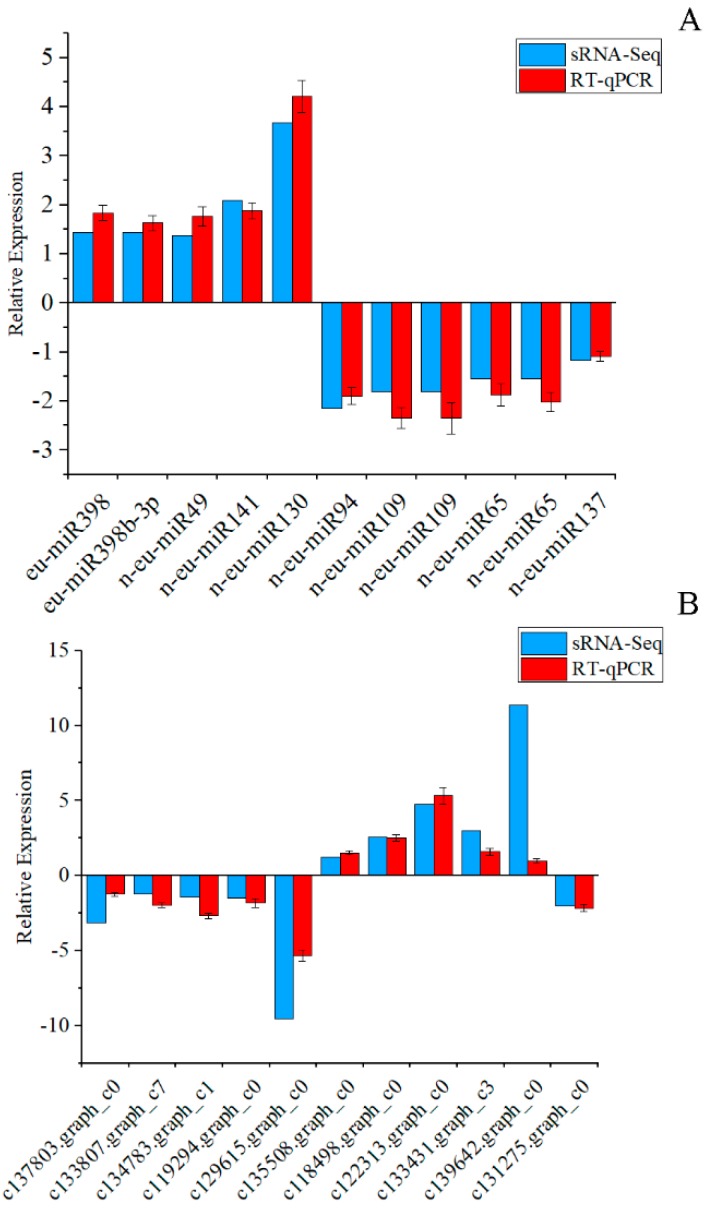
Validation of the expression of miRNAs (**A**) and their targets (**B**) through real-time quantitative PCR (RT-qPCR). The y-axis shows the relative expression (log_2_(fold-change)). Bars represent the standard deviation of the three biological replicates.

A total of 11 interesting miRNA-target models generated by high-throughput sequencing were validated by RT-qPCR. The results showed that the miRNA-target models had similar expression patterns between high throughput sequencing and RT-qPCR, although the levels of expression were different (Figure 8). This result showed that high-throughput sequencing for the quantification of miRNAs and mRNAs was reliable. 

**Figure 9:**
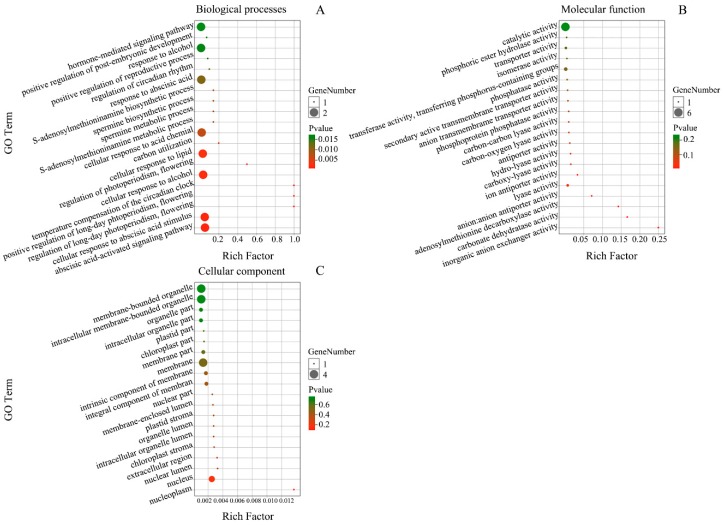
GO terms enrichment analysis of differentially expressed genes (DEGs) targeted by differentially expressed miRNAs (DEMs). (**A**) Biological process; (**B**) molecular function; (**C**) cellular component.

GO analysis of these 37 DEGs showed that they participated in 237 biological process terms, 57 molecular function terms, and 30 cellular component terms. Among them, 43 biological process terms and six molecular function terms were significantly enriched. However, there were no significant enrichment terms in the cellular component (Figure 9, Table S13).

## 4. Discussion

Previous studies of *E. ulmoides* were mainly focused on pharmacology, transcriptional and genomic levels [13,35,36,37,38], and comparatively few studies on miRNAs. Currently, only 149 miRNAs have been identified in *E. ulmoides* [10], which is very limited for understanding the roles of miRNAs in the various biological processes in *E. ulmoides*. In the present study, the regulatory network between miRNAs and mRNAs was performed using the Illumina system. A total of 221 miRNAs were identified, including 68 known miRNAs and 153 novel miRNAs. Most of them had low expression abundances, indicating that sRNA sequencing is an effective strategy to identify miRNAs in plants comprehensively. Furthermore, the sRNA with 24 nt was the most abundant, followed by 21 nt, which was consistent with previous studies in other plants, such as *E. ulmoides* [10], cotton [39,40] and citrus [41].

Previous studies had shown that most conserved miRNAs were more abundantly expressed in various tissues and developmental stages of plants, whereas the abundance of non-conserved miRNAs was relatively low [42,43]. As with other plants, the majority of conserved miRNAs also had high abundance in *E. ulmoides*. Additionally, miR159, miR166 and miR396 had high expression levels in the majority of plants [5,42,44]. As expected, eu-miR159 was the most abundant in *E. ulmoides*, followed by eu-miR166a-3p, and eu-miR396 had moderate prevalence. This result was basically consistent with previous research [10]. These results suggested that miR159, miR166, and miR396 may play key roles in plant growth and development and in response to biotic and abiotic stresses. MiR159 had been reported to play important roles in plant development and the defense response by regulating four MYB proteins [45,46]. Furthermore, miR159 was inferred to regulate mevalonate kinase and rubber elongating factor to participate in natural rubber biosynthesis [11]. Moveover, different members of the same miRNA family had different expression levels in various tested samples. Similar results were common in other plants. In *Sedum alfredii*, mtr-miR159a had the high expression level, sof-miR159c_1ss1CG, mtr-miR159a_R-1_1ss15AG, and far-miR159_R+1_1ss21GC had middle expression levels, and mtr-miR159a_1ss6AG, pde-miR159_R-1, mtr-miR159a_R-1_1ss1TA, mtr-miR159a_R-1_1ss2TG, and mtr-miR159b_2ss1AT20CT had low expression levels [47]. These findings suggested that one miRNA may have multiple functions in plant growth and development and secondary metabolism; different members of the same miRNA family may form a complex regulatory mechanism in plant growth and development.

In this study, a total of 31 DEMs were presumed to be involved in Eu-rubber biosynthesis (Figure 3), including one MIR172 and three MIR398 family members and 27 novel miRNAs. The functions of miR172 in flowering timing [48], nodulation in legumes [49,50,51,52], developmental phase transition [53], and response to salt stress [54] are well known. Additionally, previous studies had shown that the expression levels of miR172 were significantly negatively correlated with the content of latex and Eu-rubber [10,12]. However, the expression levels of miR172 were positively correlated with the content rate of Eu-rubber in this study. This may be due to miR172 having different regulatory mechanisms in different plants, tissues, or developmental stages. Furthermore, previous reports had indicated that miR398 responds to a range of biotic and abiotic stresses by reducing the accumulation of copper-zinc superoxide dismutase 1 (CSD1), including copper toxicity, high-intensity light, oxidative stress, ozone stress, drought and salt stress [55,56,57]. Noteworthy, three of the four members of the MIR398 family were up-regulated in HR libraries. This finding suggested that miR398 plays a crucial role in Eu-rubber biosynthesis.

Predicting and identifying targets for miRNAs is critical to our in-depth study of the functions of miRNAs. A total of 20,815 targets were predicted by psRNATarget (2017 update) software (Table S11a). Among them, 779 miRNA-target pairs were identified by degradome sequencing (Table S11b). The targets of most known miRNAs were relatively conserved among different plants, and the functions of the targets had a wide range, including transcription factors, response to biotic and abiotic stresses, signal transduction, and secondary metabolism processes. Terpenoids are generated by isopentenyl diphosphate (IPP) and its isomer dimethylallyl diphosphate (DMAPP) via the mevalonate (MVA) and methylerythritol phosphate (MEP) pathways [58]. In previous reports, some genes involved in terpenoid backbone biosynthesis were predicted to be regulated by several miRNAs. For instance, in *Panax notoginseng*, 3-hydroxy-3-methylglutaryl coenzyme A synthetase (*HMGS*) was targeted by miR5293 and miR5021, geranyl diphosphate synthase (*GPS*) was targeted by miR5163, and 1-deoxy-D-xylulose-5-phosphate synthase (*DXS*) was targeted by novel_miR_27 in *Panax notoginseng* [59] and Eu-miR91 in *E. ulmoides* [10]. In the present work, 22 unigenes involved in terpenoid backbone biosynthesis were predicted to be targeted by 34 miRNAs (Table 1). Geranylgeranyl diphosphate synthase (*GGPS*) was targeted by n-eu-miR15, which has been demonstrated by the RLM-5′ RACE assay (Figure 5). These findings suggest that miRNAs affect the accumulation of Eu-rubber via regulation of the genes involved in terpenoid backbone biosynthesis.

Most miRNAs regulate the targets at the post-transcriptional level by cleavage of targets or by inhibiting translation [60]. Comprehensive analysis of the expression of miRNAs and their targets helps to understand the function of miRNA-target modules in specific biological processes [61]. In the present study, 37 DEGs were predicted to be targeted by 22 DEMs, forming 44 miRNA-target pairs via DEGs analysis (Figure 7). Among them, more than half exhibited a negative regulatory pattern. For example, two members of MIR398 (eu-miR398b-3p and eu-miR398) were both up-regulated in HR libraries, while their target transcripts (c129815.graph_c0 and c119294.graph_c0) were down-regulated (Figure 7). However, some of them exhibited a positive regulatory pattern. For example, eu-miR172e-3p was up-regulated in HR libraries, and its target transcript (c137422.graph_c1) was also up-regulated. Nevertheless, the expression levels of miR172 were significantly negatively correlated with the content of latex [12]. However, the miRNA-target pairs involved in terpenoid backbone biosynthesis in previous researches were not found in this work, such as Eu-miR91-*DXS*, novel_miR_27-*DXS*, and miR5163- *GPS*. This may be caused by the difference in tissue or collection times of the test sample. 

In conclusion, this study firstly revealed the regulatory network of miRNAs and their targets by sRNAs, transcriptomics, and degradome sequencing in *E. ulmoides*, and it comprehensively analyzed the complex regulatory network of Eu-rubber biosynthesis. A total of 221 miRNAs and 82,065 unigenes were identified, and 31 DEMs were identified as Eu-rubber biosynthesis-related miRNAs. Furthermore, 44 miRNA-target pair (including 22 DEMs and 37 DEGs) modules were identified by comprehensive analysis of the expression profiles of miRNA and their targets. These results will advance our understanding of the miRNA-mediated molecular mechanisms of Eu-rubber biosynthesis in *E. ulmoides*.

## Figures and Tables

**Table 1:** The targets of known and novel miRNAs involved in terpenoid backbone biosynthesis.

Target ID	miRNAs	Target annotation
c146902.graph_c0	n-eu-miR39	geranylgeranyl diphosphate reductase
c128958.graph_c4	n-eu-miR85	ditrans,polycis-polyprenyl diphosphate synthase
c134980.graph_c0	n-eu-miR5	ditrans,polycis-polyprenyl diphosphate synthase
c130182.graph_c1	n-eu-miR45	1-deoxy-D-xylulose-5-phosphate reductoisomerase
c138767.graph_c0	n-eu-miR65	1-deoxy-D-xylulose-5-phosphate synthase
c132996.graph_c3	eu-miR156f-5p	prenyl protein peptidase
c148011.graph_c0	n-eu-miR76	geranylgeranyl diphosphate synthase, type II
c130198.graph_c0	eu-miR156f-3p	farnesyl diphosphate synthase
c139976.graph_c0	n-eu-miR42	geranylgeranyl diphosphate synthase, type II
c124585.graph_c0	n-eu-miR43	hydroxymethylglutaryl-CoA reductase (NADPH)
c137756.graph_c2	n-eu-miR91	phosphomevalonate kinase
c96211.graph_c0	n-eu-miR42	endopeptidase
c136111.graph_c2	n-eu-miR42; n-eu-miR152	farnesyl diphosphate synthase
c122494.graph_c0	n-eu-miR1; n-eu-miR8	acetyl-CoA C-acetyltransferase
c138663.graph_c0	eu-miR396b; n-eu-miR60	diphosphomevalonate decarboxylase
c126798.graph_c1	n-eu-miR85; n-eu-miR47	prenylcysteine α-carboxyl methylesterase
c133948.graph_c0	eu-miR396c-3p; n-eu-miR15; n-eu-miR109	geranylgeranyl diphosphate synthase, type II
c136533.graph_c5	eu-miR156b; n-eu-miR66; n-eu-miR101; n-eu-miR147	farnesyl diphosphate synthase
c121732.graph_c0	eu-miR2111a-5p; n-eu-miR1; n-eu-miR8; n-eu-miR66	1-deoxy-D-xylulose-5-phosphate synthase
c139224.graph_c1	eu-miR156b; n-eu-miR43; n-eu-miR59; n-eu-miR108	prenylcysteine oxidase / farnesylcysteine lyase
c136892.graph_c1	n-eu-miR1; n-eu-miR4; n-eu-miR94; n-eu-miR95; n-eu-miR142; n-eu-miR152	all-trans-nonaprenyl-diphosphate synthase
c137351.graph_c2	n-eu-miR1; n-eu-miR7; n-eu-miR8; n-eu-miR33; n-eu-miR43; n-eu-miR45; n-eu-miR76; n-eu-miR78; n-eu-miR152	isopentenyl-diphosphate delta-isomerase

## Supplementary Materials

The following are available online at https://www.mdpi.com/2073-4425/10/8/623/s1, Table S1: The sequences of gene-specific primers for RLM-5′ RACE analysis, Table S2: RT-qPCR primer sequences for candidate miRNAs and their targets, Table S3: Overview of the *E. ulmoides* transcriptome, Table S4: Overview of the *E. ulmoides* small RNA sequencing, Table S5: Overview of statistics of small RNA annotation, Table S6: Comparison between small RNA and the *E. ulmoides* transcriptome, Table S7: Detailed information of the known miRNAs, Table S8: The conserved miRNA families among species, Table S9: Detailed information of the novel miRNAs, Table S10: Summary of Eu-rubber biosynthesis-related miRNAs, Table S11: The predicted and identified targets of known and novel miRNAs, Table S12: Function annotation of targets of known and novel miRNAs, Table S13: GO enrichment of differentially expressed targets of DEMs.
